# Social media as a tool for scientific updating at the time of COVID pandemic: Results from a national survey in Italy

**DOI:** 10.1371/journal.pone.0238414

**Published:** 2020-09-03

**Authors:** Rita Murri, Francesco Vladimiro Segala, Pierluigi Del Vecchio, Antonella Cingolani, Eleonora Taddei, Giulia Micheli, Massimo Fantoni, COVID II Columbus Group

**Affiliations:** 1 Dipartimento di Scienze di Laboratorio e Infettivologiche, Fondazione Policlinico Universitario A. Gemelli IRCCS, Rome, Italy; 2 Dipartimento di Sicurezza e Bioetica, Università Cattolica del Sacro Cuore, Rome, Italy; 3 Catholic University of the Sacred Heart, Rome, Italy; 4 Dipartimento di Scienze Mediche e Chirurgiche, Unità Operativa Complessa di Pneumologia, Fondazione Policlinico Universitario A. Gemelli IRCCS, Rome, Italy; 5 Dipartimento Scienze dell’Invecchiamento, Neurologiche, Ortopediche e Della Testa-Collo, Fondazione Policlinico Universitario A. Gemelli IRCCS, Rome, Italy; National Institute for Infectious Diseases Lazzaro Spallanzani-IRCCS, ITALY

## Abstract

In the face of the rapid evolution of the COVID-19 pandemic, healthcare professionals on the frontline are in urgent need of frequent updates in the accomplishment of their practice. Hence, clinicians started to search for prompt, valid information on sources that are parallel to academic journals. Aim of this work is to investigate the extent of this phenomenon. We administered an anonymous online cross-sectional survey to 645 Italian clinicians. Target of the survey were all medical figures potentially involved in the management of COVID-19 cases. 369 questionnaires were returned. 19.5% (n = 72) of respondents were younger than 30 years-old; 49,3% (n = 182) worked in Infectious Diseases, Internal Medicine or Respiratory Medicine departments, 11.5% (n = 42) in Intensive Care Unit and 7.4% (n = 27) were general practitioner. 70% (n = 261) of respondents reported that their use of social media to seek medical information increased during the pandemic. 39.3% (n = 145) consistently consulted Facebook groups and 53.1% (n = 196) Whatsapp chats. 47% (n = 174) of respondents reported that information shared on social media had a consistent impact on their daily practice. In the present study, we found no difference in social media usage between age groups or medical specialties. Given the urgent need for scientific update during the present pandemic, these findings may help understanding how clinicians access new evidences and implement them in their daily practice.

## Introduction

On March the 30th, Dr M. R. attended the morning meeting of the recently born “Columbus COVID II Hospital”. During the summit, a message was read from a Whatsapp chat held by clinicians working in northern Italy, where the pandemic was displaying striking proportions. In the communication, it was said that a worrying number of cases of pulmonary thromboembolism was being reported. This was a new and previously unknown observation. After the meeting, Dr M. R. came back to the ward where Mr A, a previously healthy man in his fifties, currently recovering after a mild pneumonia due to SARS-CoV-2, was about to be discharged. He complained of a mild, yet worsening dyspnea and a lumbar pain. He had normal body temperature, blood pressure and was hemodynamically stable. Arterial blood gas analysis showed pO2 87 mmHg, pCO2 34 mmHg and SatO2 96%. Keeping the recent meeting in mind, we decided to request a chest CT scan, that revealed massive pulmonary thromboembolism. Low-molecular-weight heparin (LMWH) at therapeutic dose was started, the patient improved within a few days and was then safely discharged. To what extent the recent informal communication accelerated the diagnosis is difficult to define.

The first confirmed case of SARS-CoV-2 infection in Italy was identified in Rome on January 31th, 2020. Since then, the coronavirus disease 2019 (COVID-19) pandemic has spread around the world, catching many countries unprepared to face its enormous burden [1]. At the time this article was written, Italy was among the countries with the highest number of COVID-19 cases in the world, counting more than 162.000 total confirmed cases, with approximately 600 deaths and 3.000 new diagnosis per day. Full commercial and movement restrictions had been in place on all national territory for more than three weeks [2]. National guidelines recommended an antiviral therapy based on protease inhibitors and chloroquine [3] for nearly all hospitalized patients. Available literature focused almost exclusively on the respiratory tract manifestations, but clinical practice and not peer-reviewed evidences suggested that clinical features of the disease could be more varied and umpredictable [4, 5]. By the end of July, the total number of confirmed cases and deaths raised to 246.000 and 35.000. Clinicians on the frontline thus felt compelled to search for rapidly available, yet accountable information and started to share, in turn, any relevant finding coming from their daily practice or from preliminary data analysis. Aim of this study is to investigate to what extent physicians sought information on social media and other not conventional sources.

## Methods

In order to investigate to what extent clinicians are seeking and using information coming from social media, or other sources that are parallel to the scientific literature, we designed an anonymous, voluntary questionnaire on SurveyMonkey. The questionnaire was built as a nationwide, cross-sectional survey, targeting all medical figures potentially involved in the management of COVID-19 cases. The online form included 17 questions about basic demographic characteristics, personal involvement in the SARS-COV2 pandemic, frequency of social media utilization and the perceived impact of social media in the respondent’s practice. A total of 645 Italian clinicians received the form. Data were collected from the 5^th^ to the 14^th^ of April 2020 and analyzed from the 15^th^ to the 19^th^ of the same month.

Categorical variables were reported as proportions and compared using chi-square test. Binary logistic regression was used to determine the relationship between demographical variables and self-reported impact of social media on clinical practice. Results were adjusted for demographical variables in the multivariable analysis. An a priori P-value <0.05 was considered to be significant. All statistics were conducted using IBM SPSS Statistics Version 25 (IBM Corporation, Armonk, NY).

The present study has been approved by the Fondazione Policlinico Universitario A. Gemelli Ethics Committee. Consent was obtained by written statement.

## Results

Three hundred sixty-nine questionnaires were returned. Twenty percent of respondents (n = 72) were younger than 30 years-old and 10% (n = 37) were more than 60 years-old; 21.9% (n = 81) of the respondents worked in an Infectious Diseases department before the pandemic, 27.4% (n = 101) in Internal Medicine or Respiratory Medicine, 11.5% (n = 42) in Intensive Care Unit and 7.4% (n = 27) were general practitioner. Two-hundred and twelve respondents (57.5%) answered from Central Italy (including Lazio, our region), 112 (30.4%) from Northern Italy and 39 (10.6%) from Southern Italy. Fifty-two percent of respondents (n = 191) were visiting patients with COVID-19 at least once per week, and 46.6% (n = 172) visited confirmed COVID-19 cases every day. Data about how our colleagues sought information to obtain guidance for COVID-19 medical practice are presented in Table 1. Almost 80% of respondents (n = 285) reported seeking information in peer-reviewed papers, yet an equal rate (78.4%; n = 288) recurred to personal communications from colleagues working in other Centers at least twice per week, 39.3% (n = 145) consistently consulted Facebook groups and more than the half (53.1%; n = 196) reported to use Whatsapp chats for the same purpose at least once per week. Respondents characteristics are summarized in Table 1.

**Table 1:** Respondents characteristics.

Variable	Total sample (N = 368)	Social media impact on clinical practice^a^,^c^	OR (CI) P Value
Age,y			
20–29	72 (19.6)	27 (48.2)	0.05 (-0.51–0.61) .85
30–39	109 (29.6)	52 (47.7)	0.23 (-0.26–0.73) .35
40–49	80 (21.7)	45 (56.3)	0.50 (-0.02–1.02) .006
50–59	70 (19.0)	37 (52.9)	0.27 (-0.11–0.95) .12
60+	37 (10.0)	13 (35.1)	0^b^
Position			
Anesthesiologist/Intensive Care Unit	42 (11.4)	27 (64.3)	0.70 (-0.40–1.82) .21
Surgeon	49 (13.3)	18 (36.7)	0.25 (-0.821.34) .64
Pharmacist	4 (1.08)	2 (50.0)	0.62 (-0.97–2.21) .45
Nurse	3 (0.81)	1 (33.3)	-0.04 (-1.83–1.74) .96
Infectious Diseases specialist	81 (22.0)	38 (46.9)	0.23 (-0.85–1.31) .67
Internal Medicine	92 (25)	36 (39.1)	0.03 (-1.03–1.10) .94
Public Healt doctor	20 (5.43)	10 (50.0)	0.53 (-0.60–1.72) .37
Family doctor	30 (8.15)	24 (80.0)	1.37 (0.23–2.59) .02
Pediatrician	20 (5.43)	6 (30.0)	0.15 (-1.03–1.33) .80
Pneumologist	10 (2.71)	5 (50.0)	0.24 (-1.07–1.56) .72
Psychiatrist	4 (1.08)	2 (50.0)	0.52 (-1.08–2.14) .52
Radiologist	6 (1.63)	3 (50.0)	0.42 (-1.03–1.88) .57
No position	5 (1.35)		
		2 (50.0)	0^b^
Geographical Area			
Northern Italy	112 (30.4)	49 (43.8)	-0.01 (-0.54-0-52) .44
Central Italy	212 (57.6)	104 (49.1)	0.33 (-0.15–0.27) .96
Southern Italy	39 (10.6)	17 (51.5)	0.43 (-0.68–1.55) .21
Frequency of COVID-19 cases management			
Never	108 (29.3)	44 (40.7)	-0.24 (-0.93–0.46) .51
Occasionally	69 (18.7)	32 (46.4)	-0.06 (-0.76–0.64) .87
Once a week	19 (5.16)	8 (42.1)	0^b^
Everyday	172.(46.7)	90 (52.3)	0.20 (-0.43–0.84) .54

^a^. Survey question: “How impactful are the information acquired trough social media for your daily practice?” answers: “Impactful and “Very impactful”

^b^. Set to zero because this parameter is redundant.

^c^. Missing data were not shown.

^d^. Adjusted for age, position, geographical area and frequency of COVID-19 cases management

**Fig 1:**
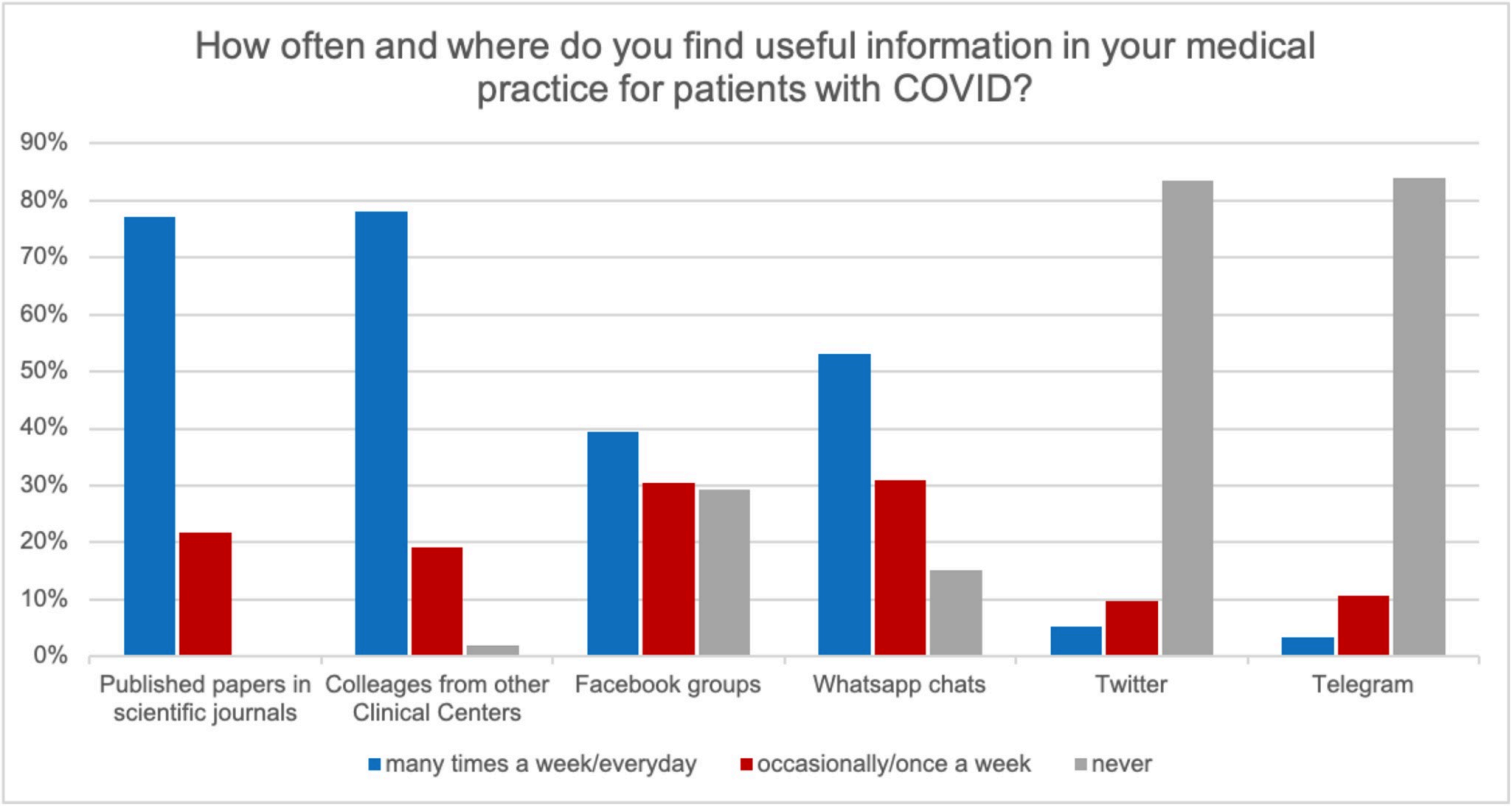
Questionnaire responses. Frequency and perceived usefulness of social media in daily practice.

Seventy percent (n = 261) of respondents reported that their use of social media to find medical information increased during the current pandemic (Fig 1). In terms of COVID-19 medical practice, information coming from social media were considered “enough” or “much” or “very much” useful by 82.9% (n = 306) of the sample. To the question “During the last week, do you think that information shared on social media had an impact on your clinical practice for patients with COVID?” 28.7% (n = 106) answered “enough” and 47.1% (n = 174) “much” or “very much”.

## Discussion

In 2016, during the Zika epidemic, a protocol for data sharing during public health emergencies was issued by the World Health Organization [6]. Currently, several academic journals are trying to meet the instances of the medical community by hosting open-access COVID sections while speeding up their peer-reviewing process. Special web pages have also been created to accelerate data sharing on this disease, such as the NEJM Coronavirus page [7], the Lancet COVID-19 Resource Centre [8], the BMJ’s Coronavirus (COVID) Hub [9], and the Cell Press Coronavirus Resource Hub [10]. Even scientific societies, foundations and consortia opened dedicated sections on their website [11].

However, although providing great opportunities, social networks create the ideal framework for misinformation to spread [12, 13]. This is particularly true in time of pandemics, when a substantial increase in demand pressure, along with poor supervision of online contents can easily lead to misinformation dissemination [14]. Alarmingly, this phenomenon has the potential to undermine trust in health institutions and programmes, especially when governments rely almost solely on empirical evidence for policy-making [15]. In fact, studies conducted during the COVID-19 pandemic showed that the rate of tweets with false or unverifiable contents may be as high as 24% [16]. Similar results were described for other pandemics, such as for 2009 H1N1 [17] and 2014 Ebola outbreaks. Interestingly, data from the Ebola crisis showed that statements that were political in nature were particularly at risk to spread misinformation [18]. However, to our knowledge, few studies have explored the role of information dissemination through social media on clinicians and other healthcare professionals.

Our survey shows that, at the time of COVID pandemic, many clinicians react to their urgent need for updates by seeking information through unconventional sources instead of academic journals publications. Data obtained from colleagues working on different centers, Facebook groups and informal Whatsapp chats seem to be highly valued and trusted. These findings may reflect the need of a more flexible, user-friendly way to seek for medical information and updates, while the current epidemic is boosting the usage of social media to access to the complex, rapidly evolving amount of evidence that is increasingly emerging from all around the world. Interestingly, 150 responders (40.7%) reported to actively share medical information via social media “often” or “everyday”. This, on one hand, is coherent with the purpose of social media themselves but, on the other hand, it entails a broader shift in how professionals conceive the access to medical information, technological advances and scientific knowledge. We believe that it is important to acknowledge this phenomenon, as well as the risk of spreading misinformation, fear or research exceptionalism, with potentially dangerous consequences for public health [5, 12, 13, 18].

We strongly suggest that, during a pandemic, academic journals implement dedicated sections for rapid communications in the form of Forum sections, Rapid responses or Comments, and reserve peer reviewing for key points as needed [19, 20], in accordance to the cited WHO protocol for data sharing during public health emergencies [6]. Facilitated focus groups on social media could be another way to encourage discussion, even though, to our knowledge, no protocols are currently available.

Our study has several limitations. First, our sample was not uniformly distributed to all medical figures involved in COVID-19 epidemic, being intensive care doctors, primary care physicians likely underrepresented. Also, few doctors from southern Italy responded to the questionnaire. Second, the results have to be interpreted with caution due to the small sample size. Finally, our findings about the impact of social media on clinical practice are based upon the personal perspective of the respondents.

## Conclusions

In conclusion, rapidly sharing information could have an invaluable impact during a pandemic such as that caused by SARS-CoV-2. Methods to promote a safe open and rapid dissemination of relevant findings, as long as new technologies capable to identify relevant information [21] could provide a substantial benefit during the ongoing and future public-health crises.

## Supporting information

S1 Data (XLSX)
